# Comparison of two commercial embryo culture media (SAGE-1 step single medium vs. G1-PLUS™/G2-PLUS™ sequential media): Influence on *in vitro* fertilization outcomes and human embryo quality

**DOI:** 10.5935/1518-0557.20180024

**Published:** 2018

**Authors:** Iratxe López-Pelayo, Javier María Gutiérrez-Romero, Ana Isabel Mangano Armada, María Mercedes Calero-Ruiz, Pablo Javier Moreno de Acevedo-Yagüe

**Affiliations:** 1 Unit for Human Reproduction. UGC Clinical Laboratory. Puerta del Mar University Hospital. Cádiz. Spain; 2 Unit for Human Reproduction. Comprenhensive Care for Woman. Puerta del Mar University Hospital. Cádiz. Spain

**Keywords:** Culture media system, human embryo quality, human embryo development, pregnancy rates, frozen embryos

## Abstract

**Objective:**

To compare embryo quality, fertilization, implantation, miscarriage and clinical pregnancy rates for embryos cultured in two different commercial culture media until D-2 or D-3.

**Methods:**

In this retrospective study, we analyzed 189 cycles performed in 2016. Metaphase II oocytes were microinjected and allocated into single medium (SAGE 1-STEP, Origio) until transferred, frozen or discarded; or, if sequential media were used, the oocytes were cultured in G1-PLUS™ (Vitrolife) up to D-2 or D-3 and in G2-PLUS™ (Vitrolife) to transfer. On the following day, the oocytes were checked for normal fertilization and on D-2 and D-3 for morphological classification. Statistical analysis was performed using the chi-square and Mann-Whitney tests in PASW Statistics 18.0.

**Results:**

The fertilization rates were 70.07% for single and 69.11% for sequential media (*p*=0.736). The mean number of embryos with high morphological quality (class A/B) was higher in the single medium than in the sequential media: D-2 [class A (190 vs. 107, *p*<0.001), B (133 vs. 118, *p*=0.018)]; D-3 [class A (40 vs. 19, *p*=0.048) but without differences in class B (40 vs. 49)]. Consequently, a higher number of embryos cultured in single medium were frozen: 197 (21.00%) vs. sequential: 102 (11.00%), *p*<0.001. No differences were found in implantation rates (30.16% vs. 25.57%, *p*=0.520), clinical pregnancy rates (55.88% vs. 41.05%, *p*=0.213), or miscarriage rates (14.29% vs. 9.52%, *p*=0.472).

**Conclusion:**

Embryo culture in single medium yields greater efficiency per cycle than in sequential media. Higher embryo quality and quantity were achieved, resulting in more frozen embryos. There were no differences in clinical pregnancy rates.

## INTRODUCTION

One of the most important steps during assisted reproductive treatment (ART) is an adequate embryo culture. Since the number of children born from ART has been increasing over the years, the question of safety of ART procedures, including *in vitro* culture, has become a prevalent public health issue. Several culture media protocols have been created to this date (Geber *et al*., 2012; Youssef *et al*., 2015). Culture media to support development of zygotes to the blastocyst stage is based either on a single medium or sequential media. A single medium is capable of supporting embryonic growth through all stages of development (Machtinger & Racowsky, 2012; Biggers & Summers, 2008; Salvaing *et al*., 2016; Zhang *et al*., 2016). These formulations incorporate all the necessary ingredients for culture in a constant concentration. In contrast, the use of sequential culture media, known as the "back to nature" approach, is a two-step formulation designed to accommodate changes in embryo metabolism before and after the compaction stage of development (Werner *et al*., 2016). This approach reflects an improved understanding of the physiological requirements for embryo growing *in vivo*.

Arguments in favor of single medium culture include practical advantages, such as reducing the number of media required in the ART laboratory, as well as less manipulation and consequently, less possibility of error (Machtinger & Racowsky, 2012). Both culture strategies have demonstrated excellent clinical outcomes. However, there is no consensus among clinical programs as to which approach is optimal, and both remain in widespread use (Werner *et al*., 2016).

There has been much controversy in the literature about the most appropriate embryo culture medium (Paternot *et al*., 2010). However, studies have not compared the same culture media (Youssef *et al*., 2015; Swain *et al*., 2016; Lemmen *et al.*, 2014). There are many media currently commercially available, each with different and often unknown compositions; on the other hand, it is unclear whether medium composition affects embryo quality and *in vitro* fertilization (IVF) or intracytoplasmic sperm injection (ICSI) success rates (Mantikou *et al.*, 2013). Maintaining optimal embryo viability during *in vitro* culture is paramount for ART success. When examining which culture medium is optimal, it is practical not only to examine the ability of a culture system to produce a given implantation rate or clinical pregnancy rate (CPR), but also to determine how many embryos (both fresh and frozen) from the entire cohort are capable of producing a live birth (Ciray *et al*., 2012; Lane & Gardner, 2007). Culture media affect embryo quality, and the transfer of poor-quality embryos results in a higher rate of miscarriage and lower ongoing CPR (Marianowski *et al*., 2016).

With the growing movement in IVF clinics to transfer fewer embryos to women, there is increasing reliance on the IVF laboratory to maximize embryo viability. Subsequently, scrutiny on the culture systems and media used to sustain the human embryo *in vitro* is justified (Sunde *et al*., 2016).

Our goal was to compare quality (class A, B, C or D), number of cells, fertilization rates, implantation rates, CPR, and miscarriage rates for embryos cultured in a single medium (SAGE 1-STEP) or in sequential media (G1-PLUS™/G2-PLUS™), both up to D-2 or D-3. We also compared cumulative outcomes of frozen embryos in both groups.

## MATERIALS AND METHODS

This retrospective study included 189 couples who underwent infertility treatment in 2016. The outcomes were compared between two commercially available embryo culture media widely used in human ART. The single medium protocol used SAGE 1-STEP with albumin solution (Origio), while the sequential media protocol used G1™ plus (Vitrolife) up to D-2 or D-3 and G2™ plus to transfer, both containing serum albumin, hyaluronan, and gentamicin (Vitrolife). We used the terms single medium and sequential media to distinguish both commercial culture media. This study was carried out in a public institution which performs oocyte retrieval from Monday to Wednesday and embryo transfer from Wednesday to Friday (D-2 and D-3). Therefore, we have compared both media system until D-2 or D-3. The couples were allocated into one of two groups (single medium or sequential media) depending on the week of oocyte retrieval. We used the same medium for all oocytes retrieved during a week, and changed the medium every week.

This study was approved by our institutional ethics committee. Consent was obtained before ovarian stimulation from all patients who met the inclusion criteria and expressed a desire to participate in the study.

### Patients

Since this study was performed in a public institution member of the SAS (Servicio Andalúz de Salud), we may assume that SAS guidelines (Servicio Andalúz de Salud, 2016) were applied, and thus we use these guidelines as inclusion criteria: maternal age ≤41 years at the time of IVF, body mass index (BMI) less than 32, anti-Müllerian hormone (AMH) level >0.5ng/mL, and baseline antral follicle count ≥5. Oocytes from 83 patients were cultured in the single medium group, while oocytes from 106 patients were cultured in the sequential media group.

### Stimulation and oocyte collection

The patients were stimulated with a gonadotropin-releasing hormone (GnRH) agonist protocol (leuprorelin acetate 0.2mL). They were desensitized with 0.2mL per day of leuprorelin acetate (Procrin, Abbott, Spain) subcutaneously. On the first few days of menstruation, if baseline levels of estradiol (E2) <50pg/mL were achieved, ovarian stimulation started with 150-225 of recombinant follicle stimulating hormone (r-hFSH, Gonal F, Merck Serono, Germany) and 75-150 HP-hMG (Menopur, Ferring, Switzerland) subcutaneously, per day. Finally, recombinant human Chorionic Gonadotropin (hCG, Ovitrelle, Merck Serono, Germany) was administered (250 µg subcutaneously) when at least two follicles had reached a mean diameter of 17mm. Oocyte retrieval was performed 36h later, followed by ICSI.

### Sperm collection

Semen was collected by masturbation from patients' partners or frozen semen from the bank. The sperm was washed with sperm-washing medium (Pure Sperm Wash, Nidacon) and then centrifuged for 10 minutes at 300g. The button was incubated for 45 minutes at 45°C with 500µL of sperm-washing medium. Then, the best motile spermatozoa were separated, taking a 400-µL aliquot.

### Fertilization, embryo culture, and evaluation of embryo development

After oocyte retrieval, cumulus-oocyte complexes were trimmed of excess cumulus cells and cultured in a 500-µL dish of G-IVF™ plus (Vitrolife). After 2h, the oocytes were stripped and the metaphase II (MII) oocytes were injected by ICSI. Then, oocytes were cultured individually, under oil, in 50-µL droplets of either medium at 37°C in an atmosphere of 6% CO_2_. The incubators were the same in both groups (Heracell™ 150, ThermoFisher Scientific). We only included in the study the oocytes that were injected by ICSI. They came mainly from couples with the male factor.

On the following day, 17-19h later, the oocytes were checked by two observers for normal fertilization by the presence of two pronuclei and two polar bodies. The fertilization rate was defined as the percentage of correctly fertilized zygotes among the total number of mature oocytes microinjected. The embryos were classified daily according to standard morphological parameters by two observers using ASEBIR (Asociación para el estudio de la biología de la reproducción) guidelines (Hurtado de Mendoza y Acosta & Cuadros Fernández, 2015). Depending on the number of cells, size of the cells, fragmentation, multinucleation, and compaction on D-3, embryo quality was classified from class A (the best) to class D (the worst). Classes A and B were transferred or cryopreserved until D-2 or D-3, class C embryos were cultured until blastocyst stage and if they were of good quality, they were vitrified. Class D or blocked embryos were discarded. We compared fertilization rates, embryo quality in D-2 and D-3 and number of cells in D-2 and D-3 between both culture media. Sequential media were renewed every day into a new drop of medium and single medium was not renewed until transfer, frozen or discarded as the manufacturers recommend.

### Cryopreservation and thawing procedures

The best embryos that were not transferred were cryopreserved using a vitrification protocol, as per described by the manufacturer (Medicult Vitrification Cooling, Origio), followed by warming (Medicult Vitrification Warming, Origio) in a closed vitrification system. We only vitrified the best embryos generated, according to the morphology criteria described in the ASEBIR guidelines. These embryos were of class A and some of the best of class B. The percentage of frozen embryos was defined as the frozen embryos among the total embryos generated in each group. We considered frozen embryo transfers done with the two media types cultured to measure cumulative CPR in 2016. In some cases, all the good quality embryos were cryopreserved (women with high response, non-receptive endometrium or some circumstances that precluded the transfer).

### Embryo recipients and transfer

On D-2 or D-3 after oocyte retrieval, the embryos were examined and two were selected for transfer. The day of the transfer depends on the number of generated embryos (<4 embryos on D-2 and ≥4 embryos on D-3). The culture media used for transfer in the single medium and sequential media protocols were SAGE 1-Step and G2™ plus, respectively. All embryo transfers were performed using a soft transfer catheter (Labotect) under transabdominal ultrasound guidance (Toshiba Aplio X6 with a Toshiba STV-GMHZ transvaginal probe). In all cases, the luteal phase was supported with vaginal progesterone (200 µg/8h) starting on the evening of the day after oocyte retrieval. Each recipient was given transdermal patches of E2 (50 µg/48h, Evopad, Janssen-Cilag, Johnson & Johnson, Belgium) on D-2 or D-3 of the cycle. Serum β-hCG was measured 14 days after transfer. Pregnancy was confirmed through vaginal ultrasonography 4 weeks after the β-hCG test. The implantation rate was defined as the number of sacs present vis-à-vis the total number of embryos transferred. The CPR was defined as the percentage of pregnancies with a fetal heartbeat per embryo transfer. We analyzed if implantation rate or CPR varied based on the culture medium used.

### Statistical analysis

We confirmed the appropriateness of nonparametric methods with the Kolmogorov-Smirnov test. Continuous variables were compared with the Mann-Whitney *U* test, and categorical variables, with the chi-square test. All analyses were performed using the PASW Statistics 18.0 software (SPSS Inc., Chicago, USA). A two-sided *p*-value <0.05 was considered significant.

## RESULTS

The number of patients, mean age and AMH were similar in both groups (Table 1).

**Table 1:** Characteristics of patients in single medium and sequential media groups.

	Single medium	Sequential media	*p*
**Number of patients**	83 (44)	106 (56)	NS
**Age (years)**	35 [30-40]	34 [29-39]	NS
**AMH (ng/mL)**	2.34 [0.46-4.22]	2.01 [0.66-3.36]	NS
**Metaphase II oocytes obtained**	686 (47)	726 (53)	NS

NS: not significant. The results are presented: number (percentage) or median [interquartile range]

The number of MII oocytes microinjected in the single medium group was similar to that in the sequential media group (686 vs. 726 respectively, *p*=0.310). We did not find differences in fertilization rates (single medium, 70.07%; sequential media, 69.11%, *p*=0.736).

We found a significant difference between the number of frozen embryos in each group [single medium, 197 (21%); sequential media, 102 (11%), *p*<0.001].

The quality of developed embryos and the number of cells at D-2 and D-3 are described in Table 2 and 3, respectively.

**Table 2:** Embryo quality according to the ASEBIR guidelines on D-2 and D-3 for embryos cultured in single medium or sequential media.

Class	No. embryos Single medium (%)	No. embryos Sequential media (%)	*p*
**D+2 **	n=486	n=472	
**A**	190 (39)	107 (23)	<0.001
**B**	133 (27)	118 (25)	0.018
**C**	154 (32)	215 (45)	NS
**D**	9 (2)	32 (7)	0.012
**D+3**	n=141	n=145	
**A**	40 (28)	19 (13)	0.048
**B**	40 (28)	49 (34)	NS
**C**	47 (34)	59 (41)	NS
**D**	14 (10)	18 (12)	NS

No: number. NS: not significant.

**Table 3:** Number of cells at D-2 and D-3 for embryos cultured in single medium or sequential media.

No. cells	No. embryos Single medium (%)	No. embryos Sequential media (%)	*p*
**D+2 **	n=486	n=472	
**<4**	72 (15)	138 (29)	<0.001
**4**	279 (57)	205 (43)	<0.001
**5**	78 (16)	64 (14)	NS
**6**	29 (6)	23 (5)	NS
**>6**	16 (3)	17 (4)	NS
Discarded*	25 (5)	12 (4)	NS
**D+3**	n=141	n=145	
**<7**	29 (20)	53 (37)	0.099
**7-8**	86 (61)	52 (36)	0.046
**9-10**	16 (11)	17 (12)	NS
**>10**	5 (4)	10 (7)	NS
Discarded*	5 (4)	13 (8)	NS

* Embryos blocked or of very bad quality. No: number. NS: not significant.

There were a high number of embryos of better quality (class A and B) with single medium vs. the sequential media: 403 vs. 293 in D-2 and D-3, but not all were frozen. Some of them were transferred or considered unsuitable for cryopreservation. The embryo utilization rates (transferred or cryopreserved from the total embryos generated) was 51.50% for single medium and 45.06% for the sequential media group, respectively.

Fewer embryos were transferred in the single medium group than in the sequential media group (126 vs. 176), but the difference was not significant (*p*=0.692). The number of transfers done was 68 in the single medium group vs. 95 in the sequential group. The morphologic score of transferred embryos were: single medium (class A: 56, class B: 59 and class C: 61 embryos); sequential media (class A: 74, class B: 36 and class C: 16 embryos). We found no differences in implantation rates (single medium, 30.16%; sequential media, 25.57%; *p*=0.520). Finally, we found no differences in CPR between the groups (single medium, 55.88%; sequential media, 41.05%; *p*=0.213). The miscarriage rate was 14.29% in the single medium group vs. 9.52% in the sequential media group (*p*=0.472). We measured the cumulative CPR adding frozen transfer done in 2016. In the single medium group, we performed 53 transfers, and 22 women achieved clinical pregnancy (41.51%), while in the sequential media group, we performed 35 transfers, and only 10 women achieved clinical pregnancy (28.57%). This corresponded to cumulative CPRs of 49.60% in the single medium group vs. 37.70% in the sequential media group (*p*=0.357).

Likewise, there were 5 miscarriages in the single medium group and 1 in the sequential media group, all in frozen transfers. The cumulative miscarriage rate was 16.90% vs. 9.61% in the single and sequential groups, respectively (*p*=0.266).

## DISCUSSION

**Figure 1:**
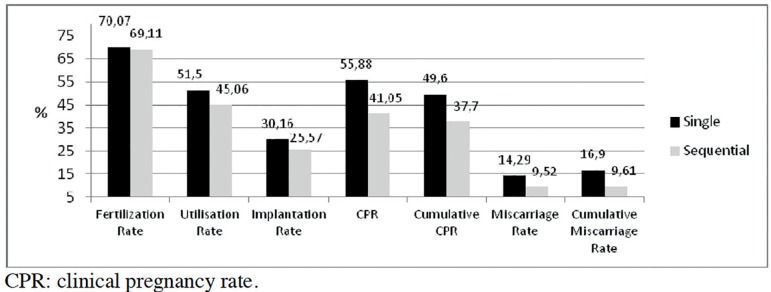
*In-vitro* fertilization outcomes comparing single vs. sequential media.

In this study, the number of patients, mean ages, and ovarian responses measured by AMH was similar in both groups; thus, we were able to compare outcomes. We did not find differences in fertilization, utilization, implantation, CPR, cumulative CPR, miscarriage or cumulative miscarriage rates (Figure 1); but the numbers of embryos produced and their quality at D-2 and D-3 were better in the single culture medium group. There has been a great difference between both culture media. We found differences in D-2 in class A, B and D and in D-3 in class A embryos. In our experience, single medium strategies are more efficient than sequential ones, but the literature remains controversial on this point (Pomeroy *et al*., 2008; Paternot *et al*., 2010; Hennings *et al*., 2016).

We found differences in the number of cells at D-2 and D-3. A large percentage of embryos cultured in the single medium group had more cells, and some of them began to compact on D-3. Compaction has been positively associated with higher implantation rates after embryo replacement on D-3 (Skiadas *et al*., 2006). Hennings *et al*. (2016), concluded that single medium strategies yield greater cell numbers, increased growth rates, and increased embryo hatching.

The main limitation of this trial is that embryo culture was performed on D-2 or D-3 and thus no information is available on later stages or the relationship between early events and blastocyst formation. But, the relationship between early events and implantation potential is available, and it plays a role in embryo selection, and thus it is important to know if these events are affected by culture media too.

Although the exact formulation of commercial media is not disclosed by manufacturers, there are some independent studies which identified the composition of some culture media used in human ART (Sfontouris *et al*., 2016; Bartmann *et al*., 2016; Morbeck *et al*., 2017). It has been documented that certain amino acids, for instance, are integral components of embryo culture media, as they are important at all stages of development and serves as the energy source needed to maintain homeostasis. Nevertheless, they break down spontaneously and release ammonia into the culture media. It has been proven that the building up of ammonia has a negative impact on embryo development (Marianowski *et al*., 2016). Morbeck *et al*. (2017) published the composition of amino acids and other components of our single medium (SAGE 1-STEP) and Bartmann *et al*. (2016) of our sequential media (G1™ plus/G2™ plus). We found subtle differences in some amino acids (single medium contains certain amino acids like Cystine, Histidine, Isoleucine, Leucine or Lysine, that are not in sequential media) and other energy substrates like glucose, lactate or piruvate that are not present in sequential media. The omission of glucose, nevertheless, seems paradoxical, because the oviduct contains significant amounts of it, and ignores the fact that media exist, in which glucose is not harmful to early preimplantation development (Biggers & Summers, 2008). Differences in composition of single and sequential media may thus contribute to the variations observed in embryo quality and development (Reed *et al*., 2009; Hentemann & Bertheussen, 2009). This could be one of the reasons for our better IVF outcomes when embryos were cultured in the single medium. Although there was no significant difference in some of the variables analyzed, the global IVF outcome was better when using the single medium. It would thus be essential for the embryology community to know the detailed composition of each medium, and there is a lack of studies comparing media and enough evidence to support or refute one specific medium (Sfontouris *et al*., 2016; Morbeck *et al*., 2017).

Our findings confirm that the use of a single medium as compared with a sequential media system has several practical advantages: a reduction in the possibility of unintentional handling errors, a reduction in staff labor and cost related to quality testing of the media, and an overall reduction in the costs of consumables. We use not-renewed single medium and the sequential media was renewed every day until the end of the embryo culture as the manufacturers recommend us. Furthermore, the role of temperature, pH, light and stress induced by pipetting was less with single medium. It requires less manipulation and consequently less incubator door opening. Wale *et al*. (2016) shows that a combination of all the above can influence the viability of human embryos and the long-term effect on the fetus.

Despite ample research in this field, no two studies to date have compared the same culture media, the same culture protocol, and the same outcome (Mantikou *et al.*, 2013; Youssef *et al*., 2015). Hence, the need for more studies on single medium vs. sequential media strategies seems to be justified in order to reach more solid conclusions regarding the comparative efficacy of these two culture approaches (Chronopoulou & Harper, 2015; Sfontouris *et al*., 2017).

The best (usually two) embryos were selected for transfer, and the morphological scores of transferred embryos differed between the two culture groups. In the sequential media group there were more class C embryos transferred - this correlated with worse quality and low implantation rates and CPR. In our trial, implantation rates, CPR and cumulative CPR were similar in both groups. Conversely, some studies concluded that culture in single medium resulted in disappointingly low implantation and pregnancy rates (Bolton *et al*., 1991; Noda *et al.,* 1994; Kleijkers *et al*., 2016). We did not find significant differences in cumulative miscarriage rates, but there were numerically more miscarriages in the single medium group.

Our results agree with previous studies that showed no advantage of single medium strategies over sequential ones for culture and development of human embryos (Sepúlveda *et al*., 2009). However, although we did not find significant differences, the number of embryos generated, the number of higher-quality embryos, and, consequently, the number of frozen embryos were higher in the single medium group. Another important aspect of our study was that our population was not selected; it comprised couples who presented to the assisted reproductive unit with fertility problems and were randomly allocated to one culture protocol, or the other depending on the week of the oocyte retrieval.

In conclusion, despite the absence of a statistically significant difference in fertilization rates, implantation rates, CPR, cumulative CPR, miscarriage rates, or cumulative miscarriage rates, we conclude that single medium culture is associated with a higher number of embryos suitable for embryo transfer or cryopreservation than culture in sequential media. Although we have demonstrated a significant effect of embryo culture media on IVF outcomes, more trials are necessary.

## References

[r1] Bartmann A, Amaral AT, Gonçalves L (2016). A descriptive study of culture media in Brazilian assisted
reproduction clinics. JBRA Assit Reprod.

[r2] Biggers JD, Summers MC (2008). Choosing a culture medium: making informed
choices. Fertil Steril.

[r3] Bolton VN, Wren ME, Parsons JH (1991). Pregnancies after in vitro fertilization and transfer of human
blastocysts. Fertil Steril.

[r4] Chronopoulou E, Harper JC (2015). IVF culture media: past, present and future. Hum Reprod Update.

[r5] Ciray HN, Aksoy T, Goktas C, Ozturk B, Bahceci M (2012). Time-lapse evaluation of human embryo development in single
versus sequential culture media-a sibling oocyte study. J Assist Reprod Genet.

[r6] Geber S, Bossi R, Guimarães F, Valle M, Sampaio M (2012). Effects of transfer of embryos independently cultured in
essential and sequential culture media on pregnancy rates in assisted
reproduction cycles. J Assist Reprod Genet.

[r7] Hennings JM, Zimmer RL, Nabli H, Davis JW, Sutovsky P, Sutovsky M, Sharpe-Timms KL (2016). Improved Murine Blastocyst Quality and Development in a Single
Culture Medium Compared to Sequential Culture Media. Reprod Sci.

[r8] Hentemann M, Bertheussen K (2009). New media for culture to blastocyst. Fertil Steril.

[r9] Hurtado de Mendoza y Acosta MV, Cuadros Fernández J, Asociación para el estudio de la biología de la
reproducción (2015). Criterios ASEBIR de valoración morfológica de oocitos,
embriones tempranos y blastocistos humanos.

[r10] Kleijkers SH, Mantikou E, Slappendel E, Consten D, van Echten-Arends J, Wetzels AM, van Wely M, Smits LJ, van Montfoort AP, Repping S, Dumoulin JC, Mastenbroek S (2016). Influence of embryo culture medium (G5 and HTF) on pregnancy and
perinatal outcome after IVF: a multicenter RCT. Hum Reprod.

[r11] Lane M, Gardner DK (2007). Embryo culture medium: which is the best?. Best Pract Res Clin Obstet Gynaecol.

[r12] Lemmen JG, Pinborg A, Rasmussen S, Ziebe S (2014). Birthweight distribution in ART singletons resulting from embryo
culture in two different culture media compared with the national
population. Hum Reprod.

[r13] Machtinger R, Racowsky C (2012). Culture systems: single step. Methods Mol Biol.

[r14] Mantikou E, Youssef MA, van Wely M, van der Veen F, Al-Inany HG, Repping S, Mastenbroek S (2013). Embryo culture media and IVF/ICSI success rates: a systematic
review. Hum Reprod Update.

[r15] Marianowski P, Dąbrowski FA, Zyguła A, Wielgoś M, Szymusik I (2016). Do We Pay Enough Attention to Culture Conditions in Context of
Perinatal Outcome after In Vitro Fertilization? Up-to-Date Literature
Review. Biomed Res Int.

[r16] Morbeck DE, Baumann NA, Oglesbee D (2017). Composition of single-step media used for human embryo
culture. Fertil Steril.

[r17] Noda Y, Goto Y, Umaoka Y, Shiotani M, Nakayama T, Mori T (1994). Culture of human embryos in alpha modification of Eagle's medium
under low oxygen tension and low illumination. Fertil Steril.

[r18] Paternot G, Debrock S, D'Hooghe TM, Spiessens C (2010). Early embryo development in a sequential versus single medium: a
randomized study. Reprod Biol Endocrinol.

[r19] Pomeroy KO, Foley S, Faber B, Moffitt DV, Johnson MD (2008). A comparison of sequential medium with non-sequential medium: do
some patients´ embryos culture better in one than in the
other?. Reprod Fertil Develop.

[r20] Reed ML, Hamic A, Thompson DJ, Caperton CL (2009). Continuous uninterrupted single medium culture without medium
renewal versus sequential media culture: a sibling embryo
study. Fertil Steril.

[r21] Salvaing J, Peynot N, Bedhane MN, Veniel S, Pellier E, Boulesteix C, Beaujean N, Daniel N, Duranthon V (2016). Assessment of 'one-step' versus 'sequential' embryo culture
conditions through embryonic genome methylation and hydroxymethylation
changes. Hum Reprod.

[r22] Servicio Andalúz de Salud (2016). Guia de reproduccion humana asistida en el sistema sanitario
público de Andalucía [Servicio Andalúz de Salud
guidelines of assisted human reproduction]. Dirección general de
asistencia sanitaria y resultados en salud.

[r23] Sepúlveda S, Garcia J, Arriaga E, Diaz J, Noriega-Portella L, Noriega-Hoces L (2009). In vitro development and pregnancy outcomes for human embryos
cultured in either a single medium or in a sequential media
system. Fertil Steril.

[r24] Sfontouris IA, Martins WP, Nastri CO, Viana IG, Navarro PA, Raine-Fenning N, van der Poel S, Rienzi L, Racowsky C (2016). Blastocyst culture using single versus sequential media in
clinical IVF: a systematic review and meta-analysis of randomized controlled
trials. J Assist Reprod Genet.

[r25] Sfontouris IA, Kolibianakis EM, Lainas GT, Petsas GK, Tarlatzis BC, Lainas TG (2017). Blastocyst Development in a Single Medium Compared to Sequential
Media: A Prospective Study With Sibling Oocytes. Reprod Sci.

[r26] Skiadas CC, Jackson KV, Racowsky C (2006). Early compaction on day 3 may be associated with increased
implantation potential. Fertil Steril.

[r27] Sunde A, Brison D, Dumoulin J, Harper J, Lundin K, Magli MC, Van den Abbeel E, Veiga A (2016). Time to take human embryo culture seriously. Hum Reprod.

[r28] Swain JE, Carrell D, Cobo A, Meseguer M, Rubio C, Smith GD (2016). Optimizing the culture environment and embryo manipulation to
help maintain embryo developmental potential. Fertil Steril.

[r29] Wale PL, Gardner DK (2016). The effects of chemical and physical factors on mammalian embryo
culture and their importance for the practice of assisted human
reproduction. Hum Reprod Update.

[r30] Werner MD, Hong KH, Franasiak JM, Forman EJ, Reda CV, Molinaro TA, Upham KM, Scott RT Jr (2016). Sequential versus Monophasic Media Impact Trial (SuMMIT): a
paired randomized controlled trial comparing a sequential media system to a
monophasic medium. Fertil Steril.

[r31] Youssef MA, Mantikou E, van Wely M, Van der Veen F, Al-Inany HG, Repping S, Mastenbroek S (2015). Culture media for human pre-implantation embryos in assisted
reproductive technology cycles. Cochrane Database Syst Rev.

[r32] Zhang H, Zheng Y, Wu Y, Ye D, Huang X (2016). A prospective randomized comparison of early embryo cleavage
kinetics between two media culture systems. Pak J Med Sci.

